# The role of the esophageal and intestinal microbiome in gastroesophageal reflux disease: past, present, and future

**DOI:** 10.3389/fimmu.2025.1558414

**Published:** 2025-02-21

**Authors:** Yipan Guan, Hongjie Cheng, Naiwei Zhang, Yanmei Cai, Qiaoyan Zhang, Xianyang Jiang, Ao Wang, Haixia Zeng, Boyi Jia

**Affiliations:** Department of Spleen and Stomach Diseases, Fangshan Traditional Medical Hospital of Beijing, Beijing, China

**Keywords:** gastroesophageal reflux disease, microbiome, microbial dysbiosis, inflammation, barrier, motility

## Abstract

Gastroesophageal reflux disease (GERD) is one of the common diseases of the digestive system, and its incidence is increasing year by year, in addition to its typical symptoms of acid reflux and heartburn affecting the quality of patients’ survival. The pathogenesis of GERD has not yet been clarified. With the development of detection technology, microbiome have been studied in depth. Normal microbiome are symbiotic with the host and can assist the host to fulfill the roles of digestion and absorption, and promote the development of the host. Dysbiosis of the microbiome forms a new internal environment, under which it may affect the development of GERD from the perspectives of molecular mechanisms: microbial activation of Toll-like receptors, microbial stimulation of cyclooxygenase-2 expression, microbial stimulation of inducible nitrous oxide synthase, and activation of the NLRP3 inflammatory vesicle; immune mechanisms; and impact on the dynamics of the lower gastrointestinal tract. This review will explore the esophageal microbiome and intestinal microbiome characteristics of GERD and the mechanisms by which dysbiotic microbiome induces GERD.

## 1 Introduction

Gastroesophageal reflux disease (GERD) is a chronic condition characterized by reflux of gastric contents into the esophagus, which can cause uncomfortable symptoms and potential complications (1). The prevalence of GERD is estimated to be 10-20% of the adult population in Western countries and ranges from 2.5 - 7.8% in Asia (2). The chronic nature of GERD leads to psychological distress, including anxiety and depression, which not only affects the quality of life of the patient symptomatically, but is also associated with a number of serious complications (3). Routine diagnostic techniques of GERD mainly include endoscopy and 24-hour pH monitoring, and the diagnosis is usually made by symptomatic and empirical qualitative examination of the patient’s condition. The initial diagnosis is often made by symptoms and empirical proton pump inhibitor (PPI) testing. Traditional therapeutic strategies include lifestyle modifications (e.g., dietary changes and weight loss), pharmacologic interventions (primarily PPIs and histamine 2 {H2} receptor antagonists), and surgical options for refractory cases, such as fundoplication (4).

Therapeutic aspects of the combined use of probiotics have a positive effect on GERD. In a clinical trial, patients with esophagitis (RE) were randomized into a test or control group, the test group received rabeprazole tablets + Streptococcus lactis MH-02, and the treatment group received rabeprazole tablets + placebo, and the results of the trial showed that patients in the test group experienced earlier symptom relief, significantly lower Gastrointestinal Symptom Rating Scale (GSRS) scores, and a longer mean time to relapse (5). Another study also examined patients with RE, with the test group taking esomeprazole + Bacillus subtilis and Enterococcus faecalis enteric capsules, and the control group taking esomeprazole + placebo, and its results showed that the time to relapse was significantly shorter in the control group than in the test group, and that the risk of relapse was lower in the test group than in the treatment group at any point in time during the 12-week follow-up period (6). The above trial results suggest that microbiomes may have a positive impact on the treatment of GERD from a more profound perspective.

This paper will summarize the structural characteristics of the esophageal and intestinal microbiome of GERD and the known possible triggering mechanisms and potential future triggers are detailed.

## 2 Esophageal microbiome

Traditionally, the esophagus was thought to have no significant microbiota, and the microbiota in the normal esophagus was thought to be derived from the oral cavity and to be variable (7–10). In 1998, Gagliardi et al. isolated the microorganism *Streptococcus viridans* from oropharyngeal cultures by traditional esophageal culture (9). With the development of next-generation sequencing technologies such as 16S rRNA gene sequencing, internal transcribed spacer region (ITS) sequencing, Polymerase Chain Reaction (PCR), Birdshot Macro-genomics, Macro-transcriptomics, Metabolomics, and Macro-proteomics Mass Spectrometry, etc., the study of esophageal microecology has been gradually improved (11). It has been found that some members of the phylum *Thick-walled Bacteria*, including *Clostridium* spp*, Fusobacterium* spp*, Megalococcus* spp, *Morgillus* spp, and *Moriella* spp, are unique microbiota of the esophageal mucosa, which are found only in the esophagus and not in the oral cavity (12–14).

### 2.1 Dominant bacterial families in the esophagus of healthy individuals

Esophageal microbiota is the dynamic community of microorganisms inhabiting the esophagus (15). Since the early 2000s, many scholars have begun to study sesophageal microbiomes using new techniques. In 2004 Pei et al. examined biopsy tissues from the normal esophagus of four adults by using wide-range 16S rDNA PCR and showed that members of six phyla: *Thick-walled Bacteria, Anaplastic Bacteria, Actinobacteria, Aspergillus, Clostridium*, and *TM7* were all represented, with *Streptococcus* spp. *(39%), Prevotella* spp. *(17%)* and *Veronella* spp. *(14%)* were the most prevalent (11). In 2012, Fillon et al. identified the esophageal microbiota in 15 individuals from children with normal esophageal mucosa, and investigated the bacterial composition by using 16S r RNA gene sequencing to identify 31 genera, of which *Streptococcus, Prevotella*, and *Veronella* spp. were the three most common (16). Macrogenome sequencing of human populations has shown that the gastroesophageal (GE) microbiome is broadly controlled by six major phyla (ibid), and *Streptococcacae, Veilonellacae*, and *Prevotellacae* have been described by other authors as the dominant bacterial phyla in the healthy esophagus by 16Sr RNA sequencing technology (12, 16, 17).

### 2.2 Esophageal microbiome in reflux

The microbiome changed with the spatial structure of the esophagus. The relative abundance of *Streptococcus* spp. increased proximally to the middle of the esophagus and then decreased significantly in the distal esophagus, with gram-negative(G-) microorganisms concentrated in the distal esophagus (18).

#### 2.2.1 Changes in esophageal microbiome by age, medicine, and diet which potentially affecting GERD

Esophageal microbiome changes with age. A study also showed that the composition of the microbiome was more stable with age, with more gram-positive (G+) bacteria and fewer G- bacteria, regardless of disease state (19). The change in microbiome toward a G+ microbiome may have a supportive effect on esophageal function.

Esophageal microbiome changes with medication. A study recruiting healthy subjects partially treated with Proton Pump Inhibitors (PPIs) showed that short-term PPI treatment increased the microbial abundance of *Streptococcaceae, Leuconostacaceae*, and *Pasteurellaceae* at the family level and at the corresponding genus level. PPIs may enhance the colonization of some probiotic species such as *Streptococcus thermophilus* and other species present in the multi-strain probiotic (20). Another study in patients with non-erosive reflux disease (NERD), esophagitis (RE) and Barrett’s esophagus (BE) found that PPIs use was associated with a reduction in Bacteroidetes in NERD and RE (19). In another study, esophageal biopsies performed before and after 8 weeks of PPIs treatment showed a significant decrease in G- *Clostridium* spp. species and an increase in G+ Clostridia (*Clostridiaceae* and *Lacertidae* sp*ecies*) and *Actinobacteriaceae* (*Micrococcaceae* and *Actinobacteriaceae* sp*ecies*) (18). This implies that acid suppression by PPIs alters the survival environment of the microbiome in favor of G+ bacteria that prefer high pH environments, thereby altering the esophageal microbiota. Therefore, PPIs may have a positive impact on the treatment of GERD in terms of directly reducing chemical damage and improving the esophageal microenvironment to reduce the inflammatory response.

Potassium-Competitive Acid Blocker (P-CAB): have an effect on oral microbiome. In a randomized trial, patients with laryngopharyngeal reflux disease (LPRD) were enrolled were given oral vonoprazan and saliva specimens were collected before and after treatment, and *Neisseria, Burkholderia*, and *Leptospira* were found to be more prevalent in the LPRD group than in the post-LRPD group. In contrast, the LPRD group had a lower abundance of Fulminant *Prevotella* and unidentified negative bacteria compared to the post-LPRD group (21).

Esophageal microbiome changes with diet. In a study where subjects underwent esophageal sampling along with a validated food frequency questionnaire to quantify dietary fiber and fat intake, findings showed that increased fiber intake was significantly associated with increased relative abundance of thick-walled phyla and decreased relative abundance of overall G- bacteria, including *Prevotella, Neisseria*, and *Eikenella (* 22). Therefore, in the treatment of GERD while requiring dietary modifications makes sense for the treatment of GERD.

#### 2.2.2 Altered esophageal microbiome is strongly associated with GERD

In 2009, Yang et al. proposed that esophageal microbiome can be divided into two categories: type I microbiome for normal people is dominated by G+ taxonomic units, with Streptococcus spp. as the main bacterial taxa; type II microbiome for patients with GERD and Barrett’s esophagus is dominated by G- taxonomic units, including *Weyoungerella* spp., *Prevotella, Haemophilus, Campylobacter, Clostridium*, and *Actinomyces, etc.*, and his study found high exposure to type II microbiota in GERD (23). Similarly, Park found that esophageal microbiome of NERD patients was most commonly dominated by type II microbiome at the phylum level in the phyla *Thick-walled Bacteria, Aspergillus* and *Mycobacterium (* 24). *Fusobacterium, Neisseria* and *Veilonella* were commonly detected in patients with RE and BE. Blackett et al. found increased abundance of *Campylobacter* in GERD patients (25).

The ratio of G+ to G- changes in the GERD esophagus. Liu et al. By comparing the esophageal microbiome of patients in 3 groups: normal esophagus, RE, and Barrett, they found that Streptococcus spp. had a slightly higher proportion in the normal group than in the RE or BE groups, and that the esophageal microbiome in the RE/BE state was highlighted by an increased proportion of G- bacteria (26). In the Zhou trial, the composition of the microbiome of NERD patients was characterized by higher levels of Proteobacteria and Bacteroidetes, and reduced levels of the microorganisms Clostridium and Actinobacteria (27).

Overall, the dysbiosis in GERD patients is characterized by an increase in G- bacteria.

## 3 Intestinal microbiome

Intestinal microbiome are normal microorganisms in the gut that synthesize a variety of vitamins essential for human growth and development, participate in glucose metabolism and protein metabolism, maintain normal intestinal physiological functions, and antagonize the colonization of pathogenic microorganisms (28). The intestinal microbiome affects the systemic metabolism by influencing intestinal nutrient absorption and catabolism, etc. (29), which in turn affects the systemic immune and inflammatory status, and has a significant impact on the progression of disease (30). Blackett et al. (25) found that the intestinal microbiome has 128 phylotypes, which belong to 8 phyla, of which the dominant microbiome accounts for 5 phyla, which are *Thick-walled phyla*, *Actinobacteria phylum*, *Anaplasmomycetes, Clostridium* and *Methanobacteria phylum*. Based on their association with the host, the intestinal microbiome are divided into commensal, conditionally pathogenic and pathogenic bacteria (31). Several experiments have shown that *bifidobacteria, lactobacilli*, and *Streptococcus pepticus* predominate in the intestinal microbiome of healthy subjects.

### 3.1 GERD affects intestinal microbiome

GERD patients have a decreased number of commensal bacteria and an increased number of conditionally pathogenic and pathogenic bacteria in their intestinal microbiome. A retrospectively analyzed study, by comparing the intestinal microbiome between patients with GERD and healthy subjects, found that the abundance of microorganisms such as *Desulfovibrioides*, *Halobacterium* sp*ecies*, and *Sphingobacterium* was higher in patients with GERD, and that microorganisms such as *Lactobacillus* intestinalis and *Streptococcus pepticus* were in higher abundance in a control group made up of healthy subjects (32). Another randomized trial, comparing LPRD patients with healthy subjects, found that there were significant differences in the structure of the intestinal microbiome between the two groups. Not only was the relative abundance of *Actinobacteria* phylum in the LPRD group significantly higher than that of the healthy control group, but also the genera of *Rhodobacteriaceae*, and *Collins’* spp. which belong to the same phylum of *Actinobacteria*, were enriched in the LPRD, and it was also found that *Streptococcus* spp.*, Prevotella* sp*ecies*, and *Clostridium* spp. were enriched in the LPRD group (33). Using GERD patients as the study group and selecting healthy volunteers in the same period as the control group, it was found that the number of fecal *E. coli* and *Enterococcus* spp. in the test group was higher than that in the control group, and the number of *Lactobacillus* spp., *Bacteroidetes* spp. and *Bifidobacterium* spp. in the test group was lower than the control group (34). *Bifidobacteria* and *Lactobacillus* counts were significantly higher in non-GERD patients than in GERD patients, while *Staphylococcus* and *E. coli* counts were significantly lower than in GERD patients (35).

### 3.2 Changes in intestinal microbiome with GERD treatment

In patients with GERD, the relief of clinical symptoms is accompanied by changes in the intestinal microbiome, the number of commensal bacteria rises significantly after treatment. In a randomized controlled study, GERD patients were randomly grouped, the test group was given PPI+Moxabilis, and the control group was given PPI, and the within-group comparison revealed that the number of *Escherichia coli* and *Staphylococcus* spp. decreased, and the number of *bifidobacteria* and *Lactobacillus* spp. increased in the two groups, which also revealed that the clinical remission rate of the test group was higher than that of the control group, and the number of *bifidobacteria* and *Lactobacillus* spp. was higher than that of the control group, and the number of *Escherichia* and *Staphylococcus* counts were lower than the control group (36). Another randomized controlled study also divided GERD patients into 2 groups, the control group was treated with western medicine, and the treatment group was given western medicine + Chinese herbal medicine compound, and the results of the treatment showed that the symptomatic relief of the 2 groups was accompanied by an increase in the number of *Lactobacillus* and *Bifidobacterium* in the intestinal tract and a decrease in the number of *Staphylococcus* in the intestinal tract compared with that of the control group before the treatment (37). The phenomenon of improvement of symptoms and change of microbiome was also found in another trial, the results of this study showed that the clinical symptoms of patients in both groups improved significantly after treatment, and the numbers of *Enterobacteriaceae* and *Enterococcus* were lower than before treatment, and the numbers of *Lactobacillus* and *Bifidobacterium* microbiome were higher than before treatment (38).

## 4 Mechanisms of dysbiosis on GERD

**Figure 1:**
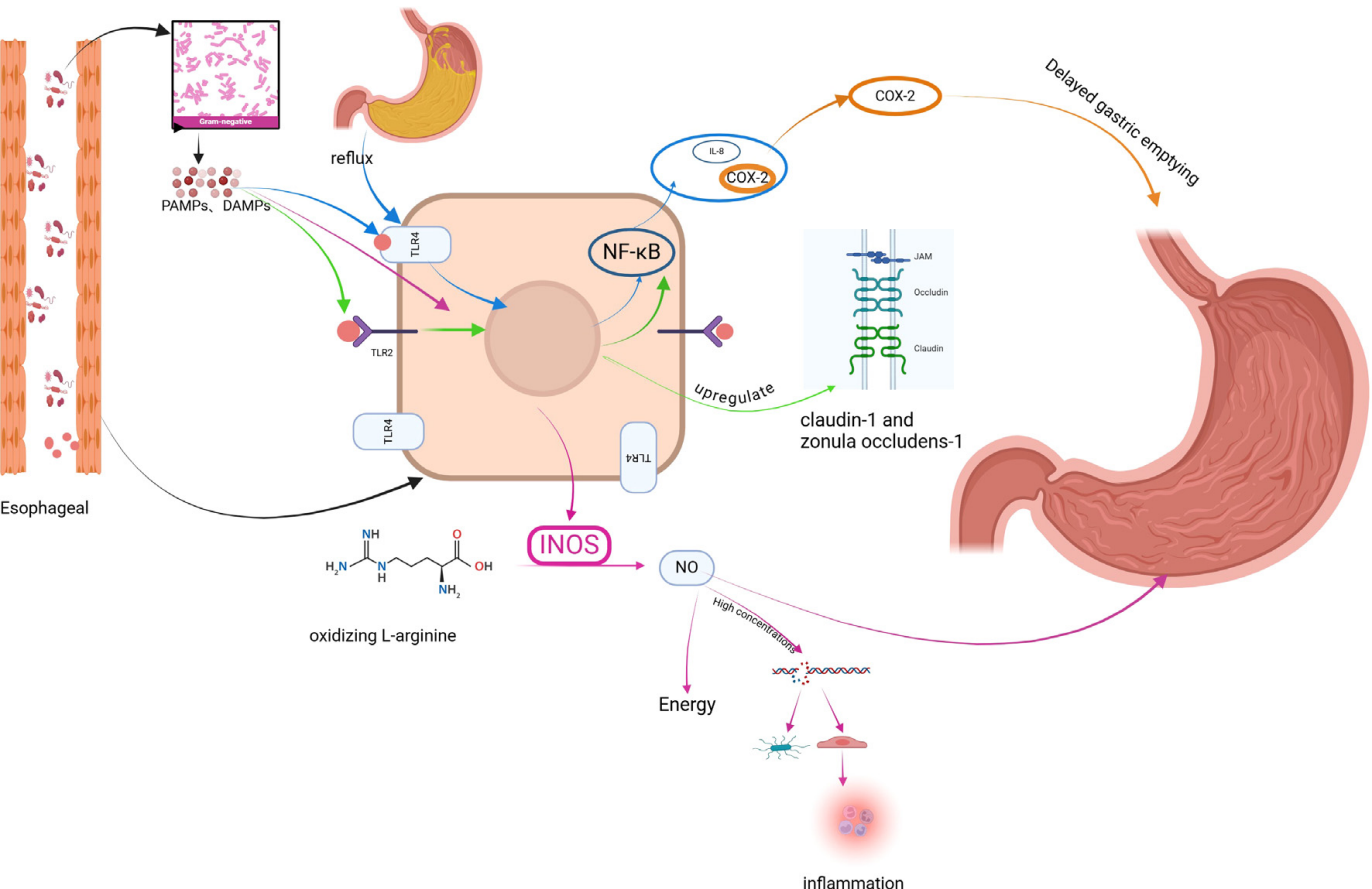
Mechanism of GERD induced by dysbiosis of esophageal microbiome. Figure created with BioRender.com.

Ecological dysbiosis is an abnormal state of the microbial ecosystem in the host (39). Dysbiosis may be one of the environmental factors contributing to the etiology of GERD (23) (**Figure 1**).

### 4.1 Activation of Toll-like receptors by dysbiotic microbiome affects esophageal barrier structure, epithelial repair, and triggers inflammatory responses involved in GERD

G- bacteria are key producers of lipopolysaccharide (LPS), which abnormally activates a variety of Toll-like receptors (TLRs) leading to epithelial barrier dysfunction and inflammatory responses leading to GERD.

TLRs are pattern recognition receptors expressed by immune cells and epithelial cells that assist the host in differentiating between pathogenic and commensal microorganisms by recognizing pathogen-associated molecular patterns (PAMPs), conserved structures specific to pathogenic and non-pathogenic microorganisms, or damage-associated molecular patterns (DAMPs) (40). Activation of TLRs exerts phagocytosis, inflammatory cytokine release, and complement activation (41, 42). When TLRs recognize PAMPs expressed by LPS, there is activation of downstream transcription factors that regulate cytokine gene expression of NF-κB, generating an inflammatory response leading to GERD.

TLR2 is widely activated in GERD patients, and activated TLR2 regulates the epithelial barrier, epithelial cell proliferation, and inflammatory responses of the body, which in turn are involved in the development of GERD. Compared with normal esophageal epithelium, TLR2 mRNA expression was increased in inflammatory cells and epithelial cells in biopsies from patients with GERD (43), and TLR2 also recognized a variety of PAMPs expressed by dysbiotic colonies (44, 45). In normal esophageal epithelium, moderately activated TLR2 up-regulated the tight junction complexes kelotanin-1 and occluding zona pellucida-1, which enhanced the function of the esophageal epithelial barrier (46). Normal G-stimulation of TLR2 is beneficial to the enhancement of esophageal barrier function, but activation of the NF-κB pathway through other pathways leads to esophageal barrier dysfunction when the microbiome is dysbiotic. Experiments have found that overexpression of the NF-κB subunit and NF-κB target genes in esophageal tissues of mice with GERD and down-regulation of the tight junction complexes, claudin1 and claudin4, resulted in esophageal barrier dysfunction in the mice. Esophageal barrier dysfunction (47). TLR2 agonists significantly increase the proliferation of epithelial cell lines through multiple protein kinase pathways, and such experimental results were further confirmed by experiments in TLR2-deficient mice, where epithelial value-added was reduced in TLR2-deficient mice (46), and this effect would potentially impact on esophageal mucosal injury and repair.

A prospective study analyzing esophageal microbiology in patients with GER found that patients with GER symptoms exhibited significantly higher TLR2 expression, reduced claudin-1 expression, and dilated intercellular spaces (DIS). *In vitro*, exposure of human esophageal epithelial cells to LES significantly upregulated TLR2 expression and downregulated claudin-1 and DIS expression. These effects can be mediated by blocking TLR2. Thus, enriched G- in patients with GER symptoms may induce esophageal barrier dysfunction via the LPS-TLR2-IL-6-claudin-1-DIS pathway (48).

TLR4 expression is increased in patients with GERD, triggering an inflammatory response involved in the further development of GERD.TLR4 expression in normal squamous epithelial samples is mainly confined to the basal layer of the squamous epithelium, which routinely may not come into contact with PAMPs expressed by the esophageal microbiota, and reflux increases TLR4 expression (49). TLR4 expression is increased (1.9-fold) in the squamous epithelium of patients with RE compared to normal esophageal squamous epithelium (50). Epithelial myofibroblasts from GERD patients were found to activate TLR4 upon acid and LPS stimulation, which in turn activates the downstream NF-κB inflammatory pathway and promotes the secretion of inflammatory factors IL-6 and IL-8 (51).

### 4.2 Dysbiotic microbiome stimulates cyclooxygenase-2 expression, affects gastric emptying, and induces GERD

Cyclooxygenase-2 (COX-2) is the rate-limiting enzyme that catalyzes the initiating step in the metabolism of arachidonic acid to prostaglandin H2 and is a precursor to prostaglandins such as prostaglandins, thromboxanes, and prostacyclins, which act as autocrine and paracrine lipid mediators in the maintenance of local homeostasis by mediating vascular function, wound healing, and inflammation (52). Dysbiotic colonies with their LPS-induced COX - 2 expression may mediate the development of GERD, a study treated rats with LPS as well as different prostaglandins and COX - 2 inhibitors and showed that the use of COX - 2 inhibitors blocked LPS-induced delayed gastric emptying, which is a possible risk factor for GERD (53, 54). Delayed gastric emptying dilates the stomach, making more food available for reflux into the esophagus. This also produces transient lower esophageal sphincter relaxation, which facilitates GERD (55).

### 4.3 Dysbiosis leads to overexpression of inducible nitric oxide synthase affecting LES function and inducing GERD

Inducible Nitric Oxide Synthase (iNOS) is an enzyme that produces nitric oxide (NO) by oxidizing L -arginine. Under the action of appropriate stimulating factors, almost any type of cell can be induced and thus express iNOS, and LPS is one of the inducing factors. Compared to normal esophagus, iNOS is overexpressed in BE (56, 57). iNOS-produced NO induces LES relaxation leading to the development of GERD, and in a mouse model of sepsis infected with lipopolysaccharides, the LES releases INOS causing impaired LES contraction, which can be blocked by utilizing NOS inhibitors (58). In addition, NO can damage pathogenic and host cells by affecting cellular energy production (59), forming inflammatory free radicals and causing DNA rupture (56, 60), leading to cell necrosis, dysfunction, and inflammatory responses that lead to GERD.

### 4.4 Activation of NLRP3 inflammatory vesicles by dysbiotic microbiome triggers inflammatory response and cell necrosis, inducing GERD

Inflammatory vesicles are expressed by epithelial and immune cells. LPS expressed by G- dysbiotic colonies both initiates and activates the NLRP3 inflammasome and leads to downstream production of IL-1 β and IL-18. The NLRP3 inflammasome triggers an immune response in due course, causing cellular pyroptosis (61). Inflammatory response, cellular pyropoiesis is a potential mechanism for the development of GERD.

### 4.5 Dysbiosis leads to abnormal immune function of the body and inflammatory response triggers GERD

NF⁃κB is an important transcriptional regulator associated with inflammation in cells and is usually inactivated, and colony dysbiosis can activate NF-KB, which in turn encodes many pro-inflammatory genes (62). In RE rats, activation of the NF-κB pathway was found to result in the release of large amounts of TNF-α and IL-6 (63). Activation of NF-κB pathway signaling initiates the transcriptional release of IL-6, TNF-α, etc., and promotes the polarization of M1 macrophages (64, 65). M1 macrophages also release a large amount of pro-inflammatory factors, such as TNF-α, IL-1β and other pro-inflammatory factors, which can cause peripheral and central sensitization, and then enhance the esophagus’s susceptibility to a variety of reflux stimuli. sensitization of the esophagus to various reflux stimuli, and symptoms such as reflux and heartburn are manifested.

Inflammatory response induced by microbiome is a possible cause of GERD. Many studies have found that reflux does not directly damage the esophageal epithelium, but stimulates the secretion of inflammatory chemokines by the epithelial cells, which induces proliferative changes in the epithelium and recruitment of inflammatory cells such as T lymphocytes, which in turn leads to the damage of the esophageal epithelium. A study reported that histological abnormalities were first observed in the esophageal tissues of rats with reflux esophagitis, and found that the recruitment of inflammatory cells and the expression of inflammatory chemokines within the esophageal mucosal epithelium were significantly earlier than the macroscopically or microscopically visible esophageal epithelial cell injury (66). In *in vitro* studies, transient exposure of human esophageal squamous epithelial cells to acidic bile salt solution did not result in epithelial cell necrosis, but rather promoted cellular secretion of IL-8 and IL-1β, which induced recruitment of lymphocytes and neutrophils further leading to epithelial cell necrosis (66). In addition, it has been found that inflammation can induce GERD by decreasing the function of the upper laryngeal-esophageal sphincter and pharyngeal-esophageal sphincter receptors (67).

Bacterial colonies stimulate the production of pro-inflammatory cytokines through multiple effector pathways, including the formation of inflammatory vesicles and nuclear factor-κB (NF-κB), stress kinases, and interferon regulatory factor (IRF); genotoxins released by bacteria, such as reactive oxygen species (ROS), reactive nitrogen species (RNS), and hydrogen sulfide (H2S), may have a direct cytotoxic effect (68); and microbial metabolites, such as short chain fatty acids (SCFAs) or lipopolysaccharides (LPS), can modulate immune cells.

### 4.6 Bacterial microbiome affecting lower GI tract dynamics may indirectly trigger GERD

The microbiome may induce reflux by affecting the lower GI tract dynamics, which in turn affects emptying. Lower GI pressure plays an important role in the pathogenesis of GERD. It has been shown that lower GI pressure is significantly elevated in patients with GERD compared to healthy subjects, which may be a major factor in the development of GERD (69, 70). It has also been suggested that delayed emptying of the lower GI tract in one-half of GERD patients leads to an increase in intragastric pressure, and that a decrease in barrier pressure (the difference between the lower sphincter pressure and the intragastric pressure) is one of the factors contributing to the pathogenesis of GERD (71). A study found a higher prevalence of small intestinal bacterial overgrowth (SIBO) in patients with RE (72) and SIOB is associated with intestinal motility failure (73, 74). One trial found that intestinal microbiome promotes circulation and distal spread of migrating motility complexes (MMCs) in the interdigestive phase, as well as intestinal transport during and after feeding (75, 76). There are also trials demonstrating the important role of *Lactobacillus acidophilus* and *Bifidobacterium bifidum* in promoting the migration of MMCs and facilitating small intestinal transit, lower GI motility (77).

### 4.7 The products of microbiome (SCFAs) indirectly protect the esophagus from multiple pathways and have potential for the treatment of GERD

**Figure 2:**
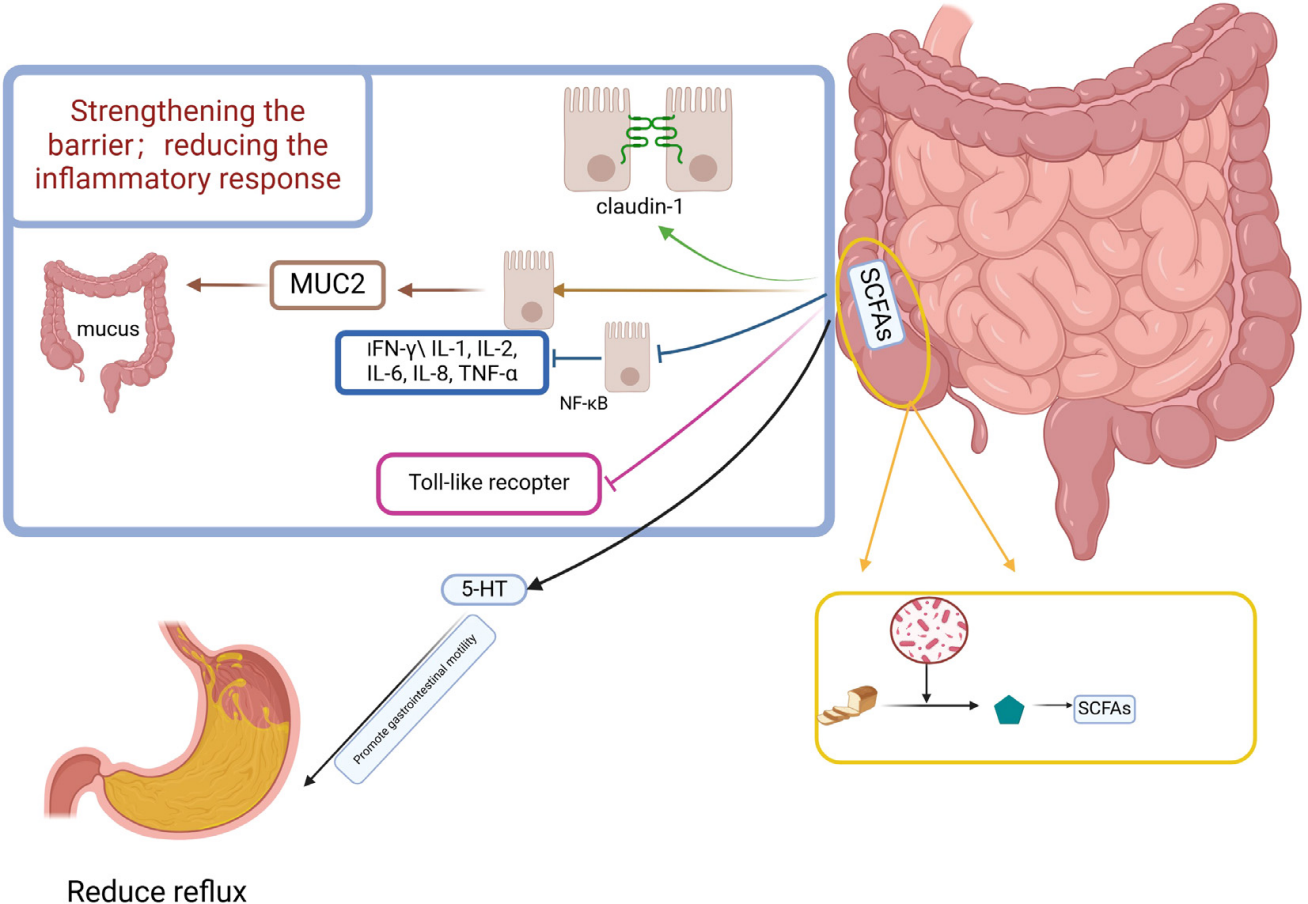
Potential mechanism of esophageal protection against reflux reduction by short-chain fatty acids. Figure created with BioRender.com.

Microorganisms in the colon are capable of fermenting indigestible food components and consuming prebiotics to produce short-chain fatty acids (SCFAs) (78, 79) The relationship between SCFAs and GERD is not yet clear, but known studies have found that SCFAs have some potential for treating GERD (**Figure 2**).

#### 4.7.1 SCFAs regulate gastrointestinal motility

Bowel motility influences GERD, and SCFAs have been suggested in several studies to influence bowel motility. Fecal SCFAs concentrations are low in patients with constipated IBS (IBS-C) and high in patients with diarrheal IBS (IBS-D) (80). SCFAs promote the production of 5-Hydroxytryptamine affecting gastrointestinal motility, enterochromaffin (EC) cells sense SCFAs and produce 5-HT, which promotes gastrointestinal motility through activation of 5-HT4 expressed on enteric neuron receptors to promote gastrointestinal motility (81); 5-HT signaling system is very important in visceral hypersensitivity, and many scholars believe that 5-HT ergic neurons are the underlying structure for the regulation of visceral sensorimotor and autonomic functions (71, 82).A randomized trial found that serum levels of 5-HT were higher in GERD patients than in healthy subjects. SCFA cells sense SCFAs and produce 5-HT subjects (83). SCFAs may affect ileal motor function after colo-ileal reflux by causing long-duration contractions and discrete clusters of contractions (84), which can directly stimulate ileal and colonic smooth muscle contractility (85). SCFAs affect emptying by influencing gastrointestinal motility, which can have an impact on reflux symptoms in GERD.

#### 4.7.2 Increase in SCFAs enhances barrier function and mucus secretion of epithelial cells

The three defense barriers of the esophageal mucosa (86), among them are the pre-epithelial barrier consisting of the mucus layer and bicarbonate, etc., and the epithelial barrier and post-epithelial barrier of the second layer of the intercellular and apical linking complexes constituting the tight junction (TJ).

SCFAs induce redistribution of the tight junction protein occludin and the closed-loop mini-loop protein ZO - 1 in the cell membrane (87–89). In monolayer differentiated intestinal epithelial cells (cdx2-ECE), butyrate enhances intestinal barrier function by increasing the expression of the TJ protein claudin- 1 (90). An experiment in an *in vitro* model of a porcine intestinal epithelial cell line (IPEC-J2) found that butyrate attenuated the negative effects of LPS on epithelial integrity while selectively up-regulating TJ protein expression (91). Another animal experiment caused changes in the colony structure of mice by altering their feeding regimen, resulting in increased production of SCFAs, and found that multiple aspects of mucus and epithelial barrier integrity were enhanced (92). SCFAs also stimulated the expression of the MUC2 gene, which resulted in an increase in mucus volume (93, 94).

There is no clear evidence that SCFAs can affect esophageal barrier function, but production of SCFAs in the colon is completely and rapidly absorbed by colonic cells and circulates systemically through the portal vein (95).

The modulation of esophageal defenses after the absorption of SCFAs into the bloodstream needs to be further studied and explored.

#### 4.7.3 SCFAs may alleviate GERD symptoms by supporting immune homeostasis and reducing inflammation

SCFA, especially butyrate, can alter the secretion of pro-inflammatory mediators (e.g., interferon (IFN) - γ (IL-1, IL-2, IL-6, IL-8, tumor necrosis factor (TNF) - α) by a possible mechanism through inhibition of NF-κB in intestinal epithelial cells (96, 97). Activation of G protein-coupled receptors (GPRs) on immune cell membranes by butyric acid leads to an increase in cytoplasmic calcium levels, and an increase in calcium concentration leads to the activation of NLRP3 inflammatory vesicles and subsequent activation of caspase - 1. Activated caspase - 1 converts pro - IL - 18 to IL - 18 which promotes epithelial repair (98), but in there are also data to support that IL-18 contributes to intestinal inflammation (98). SCFAs also inhibit the release of pro-inflammatory cytokines from intestinal epithelial cells induced by TLR activators such as LPS (99). SCFAs alleviate the symptoms of GERD by reducing the inflammatory response through their role in supporting immune homeostasis (100, 101).

### 4.8 Bacteriocins and polysaccharides protect the esophagus from multiple perspectives

Bacteriocins are ribosome-derived peptides produced by microorganisms that reside in the gastrointestinal tract and are thought to inhibit competitive microbiome. Bacteriocins in humans are thought to maintain barrier function, participate in immunomodulation, and have direct anti-microbial activity (102). Recent studies have reported that the size and nature of bacteriocins allow them to cross this intestinal blood barrier (103). Size and charge help bacteriocins to cross cell membranes and barriers and play important roles in different physiological mechanisms. Bacteriocins in the gastrointestinal tract also have specific and potent antimicrobial properties (104, 105). This antimicrobial property makes it essential in maintaining and influencing the composition of the local microbiome. It has been found in Anabaena fragilis and has been associated with the synthesis of metabolites with immunomodulatory properties. Polysaccharide A (PSA) produced by this bacterium has been shown to modulate the immune response by promoting the production of anti-inflammatory cytokines (106).

Polysaccharides (PSs) have a wide range of pharmacological activities, including modulation of immune function (107), antioxidant effects (108), anti-inflammatory properties (109), and gastrointestinal health benefits (110). PSs modulate the intestinal microbiota and have anti-inflammatory and antioxidant potential (111, 112). PSs have a positive effect on Gastroesophageal reflux (GR), and dibasic sodium alginate has emerged as a promising therapeutic option, because in addition to protecting the esophageal mucosa and limiting gastric reflux into the esophagus, they also adhere to the gastric mucosa, protecting it and promoting its repair (113). PSs regulate gastric juice secretion, increase mucus production, enhance antioxidant capacity, reduce inflammation (114, 115), and act as probiotics in the gut microbiota and are involved in protein regulation, which ensures the maintenance of barrier function and mucin production.

## 5 Discussion

Normal microbiome and the human body to live in harmony, through the microbiome of digestion, decomposition, promote the absorption of the role of the host better, the host can also provide a good environment for its habitat. Bacterial microbiome is an intelligent living organism, its existence for the organism in the end is symbiotic beneficial bacteria or harmful bacteria with toxicity, such a delineation of the boundaries should take into account not only the characteristics of the bacteria themselves, the composition of the microbial ratio, but also should take into account the host’s own functional conditions, assuming that the purpose of our medicine is to guide the adjustment of the bacterial microbiome, the use of the bacterial microbiome, so as to let it give play to its own complex, multi-faceted and subtle functions, through the treatment of disease in this way may be more comprehensive. treating the disease in this way may be more comprehensive. Understanding the microbiome as much as possible is only the first step, and the ultimate goal is to harmonize the symbiosis of good people and microorganisms.

The effects of microbiome and its metabolites on body functions are complex, and in many studies we have found that the improvement of symptoms before and after treatment is accompanied by a certain change in the microbiome structure, a phenomenon that leads us to think further about the question of whether a drug is effective by targeting a certain stage of the physiological/pathological response directly, or whether a drug is effective by affecting certain signals through changes in the internal environment affecting the structure of the microbiome, or both. Or both. Current basic research in pharmacology is mostly devoted to the effects of pathways activated by specific drugs, but the role played by drugs in the human microbiome is multifaceted. If the human microbiome can be transplanted in experimental animals, in addition to the study of the direct effect of drugs can play, but also to further observe the effect of drugs on the microbiome, the microbiome on the body.

Based on the existing research, we can clearly see the interaction between microbiome and GERD, and there is a close relationship between microbiome and the pathogenesis of GERD. However, due to the complexity of the composition of the microbiome and its effect on the organism, future research needs to gain a deeper understanding of the esophageal and intestinal microbiome and their metabolites in GERD through basic research to understand how the microbiome and their metabolites influence the occurrence of GERD, and also in the pathogenesis of GERD, abnormalities in digestive tract dynamics are gradually being emphasized by researchers. In addition, in the pathogenesis of GERD, the abnormalities of digestive tract dynamics have gradually been emphasized by researchers, and it has been further found that the brain-gut peptide (neurotransmitter) in the “brain-gut axis” is not only related to visceral hypersensitivity, but also plays an important role in regulating gastrointestinal dynamics, and changes in the bacterial microbiome and its structure can stimulate the secretion of brain-gut peptide, so the emphasis on the bacterial microbiome in the future research will also provide therapeutic opportunities for the treatment of GERD. likewise provides new therapeutic ideas for the treatment of GERD.
